# Transmission routes of cluster 3 Tembusu virus in ducks and chickens

**DOI:** 10.1099/jgv.0.002106

**Published:** 2025-06-19

**Authors:** Luzhao Li, Dawei Yan, Dun Shuo, Xiaona Shi, Minghao Yan, Chunxiu Yuan, Qiaoyang Teng, Bangfeng Xu, Xue Pan, Monique M. van Oers, Qinfang Liu, Gorben P. Pijlman, Zejun Li

**Affiliations:** 1 Department of Avian Infectious Diseases, Shanghai Veterinary Research Institute Chinese Academy of Agricultural Sciences, Shanghai 200241, PR China; 2 Laboratory of Virology, Wageningen University & Research, 6708 PB Wageningen, Netherlands

**Keywords:** chicken, duck, flavivirus, pathogenicity, Tembusu virus, transmission route

## Abstract

Tembusu virus (TMUV) is an emerging mosquito-borne flavivirus, primarily transmitted by *Culex* spp. mosquitoes. The 2010 outbreak of TMUV in ducks revealed that the virus had acquired direct contact and aerosol transmission routes, enabling its rapid spread in duck farms. Recently, cluster 3 TMUV has increasingly been isolated from chickens, ducks and geese. In this study, we examined the pathogenicity and transmission routes of the cluster 3 TMUV Shandong 2021 (SD) strain in ducks and chickens. Our results show that TMUV SD can infect both species, but only in ducks could TMUV be detected in throat and cloacal swabs. In ducks, the virus can spread without mosquito involvement to co-housed naïve birds, demonstrating direct transmission capability. Conversely, no virus shedding and direct transmission were observed in chickens, suggesting that mosquitoes are required for virus transmission between chickens. Indeed, *Culex pipiens* mosquitoes could become infected by biting chickens infected with the TMUV SD and transmit the virus to naïve chickens. Our results, for the first time, provide direct evidence that TMUV can be transmitted by mosquitoes in a laboratory setting. Furthermore, our findings indicate that viral excretion through the respiratory tract and/or digestive tract is essential for direct contact transmission of TMUV, which is a critical factor in the epidemic spread of TMUV. These insights into the transmission dynamics of a cluster 3 TMUV emphasize the importance of effective vector control and biosecurity measures in managing and preventing outbreaks.

## Data Summary

The authors confirm that all supporting data, codes and protocols have been provided within the article or through supplementary data files.

## Introduction

Tembusu virus (TMUV) is a member of the *Flaviviridae* family and was first isolated from *Cule*x *tritaeniorhynchus* mosquitoes in Malaysia in 1955 [1]. In the early twenty-first century, Tembusu virus was detected in domestic chickens in Malaysia and ducks in Thailand [2,3]. After a decade of relative silence, TMUV suddenly reemerged, causing outbreaks in ducks across China and Southeast Asia. These outbreaks have led to significant economic losses in the duck farming industry [4,6]. TMUV infection in ducks manifests as diarrhoea, neurological symptoms in ducklings and ovarian haemorrhage and necrosis in egg-laying ducks, resulting in growth retardation and a sharp decline in egg production and, in severe cases, death [4,7]. Although TMUV primarily affects waterfowl, the range of infected hosts has been expanding, posing an increasing threat to poultry farming as a whole [8,9].

TMUV isolates can be divided into three clusters. Cluster 1 includes a single TMUV strain isolated in Thailand and two strains isolated in Malaysia [10]. Since the 2010 TMUV outbreak in ducks, most isolated strains belong to cluster 2 [10,11]. However, in 2021, TMUV was isolated from chickens with reduced egg production in Shandong and Guangxi provinces, China; these strains were grouped in cluster 3 [12]. Subsequently, cluster 3 TMUV has been isolated from chickens, ducks and geese in China [11,13, 14]. Interestingly, many early mosquito-derived TMUV strains also belong to cluster 3, and cluster 3 TMUV was isolated from ducks as early as 2002 [12]. Studying the pathogenicity of chicken-derived cluster 3 TMUV in ducks is important for understanding the cross-species transmission potential of this virus.

TMUV is primarily transmitted by *Culex* mosquitoes in the wild [15]. However, on duck farms, cluster 2 duck TMUV was able to be transmitted even during the mosquito-free winter, hinting at transmission among ducks through direct contact [16]. On the other hand, cluster 3 TMUV isolated from chickens does not transmit through direct contact among chickens [12]. This study aims to explore the pathogenicity and key transmission routes of cluster 3 TMUV in ducks and chickens, providing insights into the prevention and control of TMUV outbreaks.

## Methods

### Cells and viruses

Baby hamster Syrian kidney (BHK), chicken hepatocellular carcinoma (LMH), chicken embryo fibroblasts (DF-1), chicken macrophages (HD-11) and *Aedes albopictus* (C6/36) mosquito cells were obtained from the American Type Culture Collection. Duck embryo fibroblast (DEF) cells were derived from 11-day-old duck embryos. The Shandong 2021(SD) strain (GenBank: OM240640.1) was isolated from a chicken in 2021.

### TMUV replication in cell culture

To test the viral replication *in vitro*, DEF, LMH, DF-1, HD-11 and C6/36 cells growing in T-25 flasks were infected with TMUV at a multiplicity of infection (MOI) of 0.0001. The cells were cultured in DMEM containing 2% FBS, at 37 °C (27 °C for C6/36 cells) and 5% CO_2_ (no CO_2_ for C6/36 cells). Virus samples from the supernatant were harvested every 12 h post-infection (hpi) and subjected to virus titration on BHK cells to determine virus litres. BHK cells lack the RIG-I signalling pathway, resulting in more obvious cytopathic effects in most flavivirus infections; therefore, they are a well-established choice for TMUV titration [17,18].

### Animal experiments

To test the pathogenicity and transmissibility of the TMUV SD strain in chickens or ducks, groups of nine 12- to 15-week-old birds were inoculated i.m. with 10^3.5^ TCID_50_ of the virus in a volume of 0.2 ml. One day later, three naïve birds were introduced into isolators where the nine inoculated birds were housed to test virus transmission based on the previous TMUV transmission experiment [19]. On 3 and 5 days post-inoculation (dpi), three inoculated birds in each group were euthanized by CO_2_ inhalation, and tissues from the heart, liver, spleen, lung, kidney, duodenum, thymus gland, bursa of Fabricius**,** brain, ovary and pancreas were collected to measure viral replication. Throat and cloacal swabs were collected at 2, 3, 4 and 5 dpi to measure viral shedding. The serum samples were collected from the inoculated birds at 2, 3 and 4 dpi to measure viral replication. The serum samples were collected from the inoculated birds and contact birds at 14 dpi for antibody detection. Three uninfected birds’ serum was collected as the negative control. The presence or absence of serum antibodies in the contact group indicates whether or not the virus was transmitted as demonstrated earlier [16,19].

### Mosquito infection experiments

*Culex pipiens* mosquitoes were raised with a 10% sucrose solution and kept at 28 °C with 70%–80% relative humidity in cages under standard laboratory conditions [17]. To test the infection potential of the TMUV SD strain in mosquitoes, groups of three 1- to 2-week-old chickens were inoculated intramuscularly with 10^3.5^ TCID_50_ of the virus in a volume of 0.2 ml PBS. At 3 dpi, all three chickens were exposed to biting by 50 females of *C*. *pipiens* mosquitoes (3–5 days old, with similar body sizes) in a dark cage for 2 h. In the cage, the mosquitoes could fly freely, while the chickens were restrained. A mosquito aspirator was used to collect the mosquitoes afterwards, and blood-sucked mosquitoes were selected and transferred to a new cage to be reared as infected mosquitoes. Five blood-sucked mosquitoes were homogenized directly to test for viral copies after ingestion via quantitative real-time PCR (qPCR), with five uninfected mosquitoes serving as negative controls. At 5 and 10 days post-biting, five mosquitoes were dissected into head, thorax and abdomen sections before homogenization for viral copy testing. At 10 days post-biting, five other mosquitoes were collected for paraffin embedding.

### TMUV transmission by mosquitoes

To test the transmission efficacy of the TMUV SD strain by mosquitoes, 50 female *C*. *pipiens* mosquitoes were infected as above. After 10 dpi, each of five 1-month-old chickens was placed in a mosquito cage with five infected mosquitoes that had fasted for 1 day. As described above, the mosquitoes could fly freely, while the chickens were restrained to allow biting in the dark for 2 h at 27 °C and 80% relative humidity. Afterwards, the mosquito aspirator was used to collect the mosquitoes, and the blood engorgement status of individual mosquitoes was visually determined. Serum samples were collected from the bitten chickens at 2, 3 and 4 dpi to measure viremia.

### Virus titration and RNA copy quantification

To determine viral litres, chicken or duck organs were first weighed, and PBS was added at a 1 : 1 (ml g^−1^) ratio along with steel beads. Then, the samples were homogenized using a tissue grinder at 70 Hz for 90 s. Tissue homogenates were clarified by centrifugation at 6,000 ***g*** for 5 min at 4 °C. The resulting supernatants, both undiluted and in 10-fold serial dilutions, were used to assess viral infectivity on BHK cells by end-point dilution assay in 96-well plates. The virus titre was calculated using the Reed and Muench method and expressed as tissue culture infective dose 50% (TCID_50_) per millilitre.

The RNA copy number of TMUV SD in mosquito samples was detected by qPCR following a previously established method [20]. Briefly, whole mosquitoes or mosquito parts were homogenized as described before for organs, but using an RNase-free lysis solution in the RNeasy Plus Mini Kit (QIAGEN) instead of PBS. Total RNA was extracted using the RNeasy Plus Mini Kit according to the manufacturer’s protocol. Reverse transcription was performed using Avian Myeloblastosis Virus (AMV) reverse transcriptase (Takara Biotechnology, Dalian, China) with TMUV-specific primer (5′-CGTATGGGTTGACTGTTATCA-3′). The qPCR procedure, including the primers, probe, standard curve generation and calculation of TMUV E gene copies, was performed as previously described [20].

### TMUV blocking ELISA

To quantify TMUV-specific antibodies in duck or chicken serum, a blocking ELISA was performed as previously outlined [21]. In short, ELISA plates were first coated with ~3 µg well^−1^ of inactivated and purified TMUV strain FX2010 in 0.1 M carbonate-bicarbonate buffer (pH 9.6) and incubated overnight at 4 °C. The antigen-coated plates were then washed with PBS (pH 7.4) containing 0.05% Tween 20 (PBST), and nonspecific binding sites were blocked by adding 200 µl of blocking buffer (PBS with 10% skim milk) for 1 h at room temperature. Serum samples were diluted 10-fold in PBS and added to the wells, followed by incubation at 37 °C for 1 h. Afterwards, the wells were washed three times with PBST and incubated with monoclonal antibody (MAb) 1F5(21) (1:20) at 37 °C for 1 h. Following another three washes with PBST, HRP-conjugated goat anti-mouse IgG (1:2,000; Sigma, USA) was added, and the plates were incubated at room temperature for another hour. After three final washes with PBST, 100 µl of 3,3′,5,5′-tetramethylbenzidine (Sigma, USA) was added, and the plates were incubated at room temperature for 10 min. The reaction was stopped by adding 50 µl well^−1^ 0.1 M sulphuric acid. OD was measured at 450 nm, and the percent inhibition (PI) was calculated using the formula: PI (%) = [1 − (OD_450_ of test serum/OD_450_ of negative-control serum)] × 100%. PI values of ≥18.4% indicated positive results for TMUV seroconversion.

### Mosquito paraffin embedding

At 10 dpi, mosquitoes were fixed in 4% paraformaldehyde by submergence at 4 °C for 24 h. Then mosquitoes were placed in a tissue embedding cassette to be dehydrated using a gradient alcohol series: 75% ethanol for 4 h, 85% ethanol for 2 h, 90% ethanol for 2 h, 95% ethanol for 1 h and absolute ethanol (twice) for 30 min each, followed by alcohol-benzene, xylene and three rounds of 65 °C molten paraffin incubation, each for 1 h. The paraffin-infiltrated tissue was embedded by pouring molten paraffin into the embedding frame, positioning the tissue and cooling the frame on a −20 °C platform. Once solidified, the wax block was trimmed and sliced using a microtome into 4-µm-thick sections. The sections were floated on 40 °C water, transferred to glass slides and baked dry at 60 °C. For sealing, the baked sections were briefly dipped into molten paraffin, cooled to form a protective film and then stored at room temperature. Before further experiments, the paraffin film was melted in a 67 °C oven.

### Immunohistochemistry staining of TMUV-infected mosquitoes

This section of the process was carried out by Servicebio (China). In brief, paraffin sections were sequentially dewaxed using environmentally friendly dewaxing solutions three times, followed by hydration in anhydrous ethanol three times and rinsed with distilled water. Antigen retrieval was then performed, and the slides were allowed to cool naturally before being washed in PBS (pH 7.4). Endogenous peroxidase activity was blocked by incubating the sections in 3% hydrogen peroxide at room temperature, protected from light, followed by additional PBS washes. The tissue was then blocked with 3% BSA in PBS at room temperature for 30 min. After removing the blocking solution, the sections were incubated with the TMUV E antibody (MAb) 1F5 (1:200) solution overnight at 4 °C in a humidified chamber. The following day, the sections were washed with PBS and incubated with the HRP-labelled secondary antibody Gt X Ms IgG (H+L) HRP (Sigma-Aldrich) at room temperature for 50 min. 3,3′-Diaminobenzidine (Servicebio, China) staining was performed until a brown-yellow colour developed, followed by rinsing with tap water. The nuclei were counterstained with haematoxylin, briefly differentiated, blued and rinsed with running water. The sections were then dehydrated through graded ethanol solutions, followed by n-butanol and xylene incubation, and mounted with super-clean fast-drying sealant. Finally, the results were analysed under a white-light microscope.

## Results

### Cluster 3 Tembusu virus SD can infect ducks and be transmitted by direct contact

**Fig. 1.**
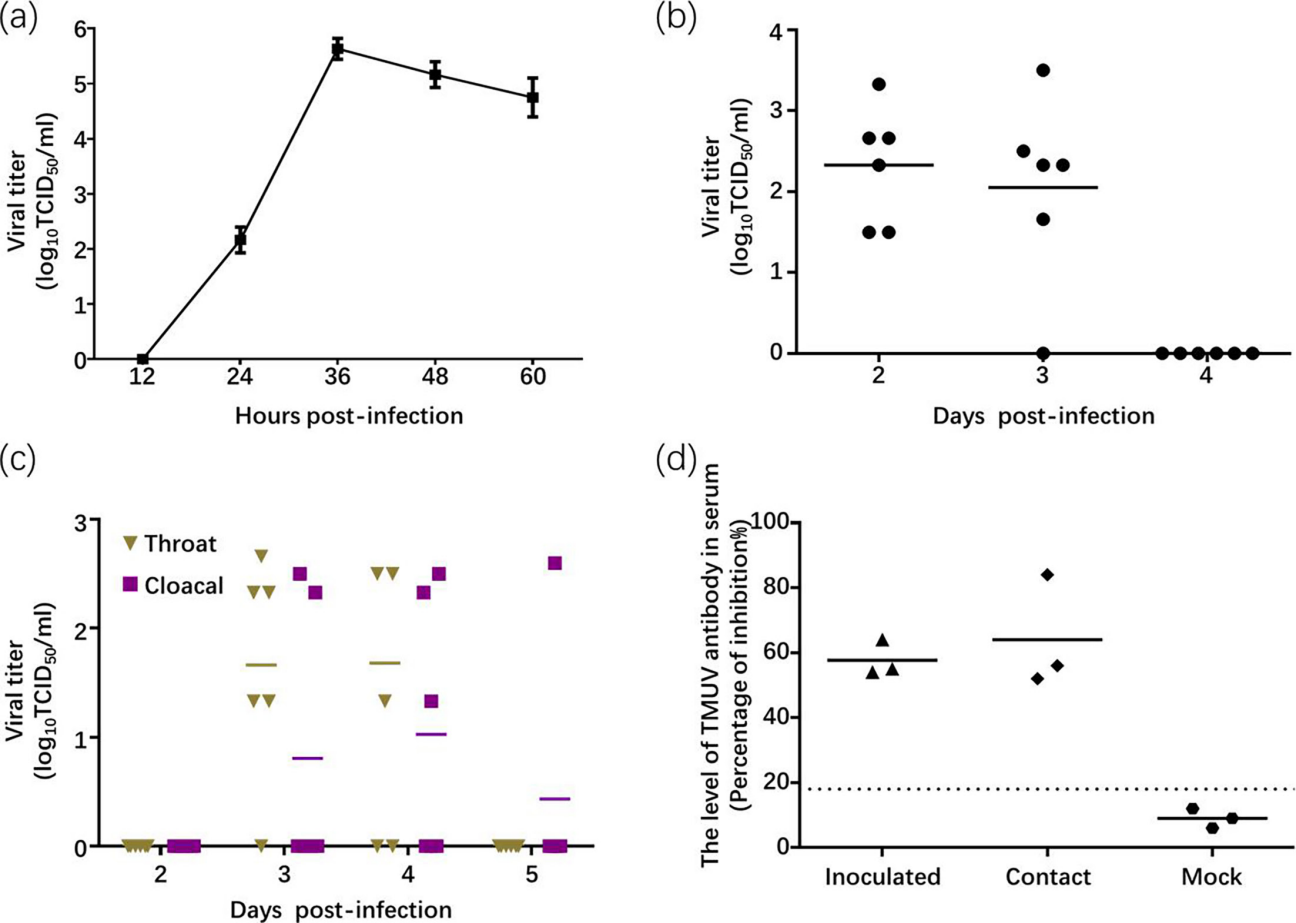
*In vitro* and *in vivo* analysis of the infectivity of cluster 3 Tembusu virus SD strain in ducks. (**a**) The replication kinetics of TMUV SD in DEF cells. Cells were infected at an MOI of 0.0001, and virus-containing supernatants were collected at different time points post-infection. Viral litres were subsequently determined by titration on BHK cells. (**b**) Viremia levels in ducks infected i.m. with 10^3.5^ TCID_50_ of TMUV SD strain. Virus litres in the blood collected at 2, 3 and 4 dpi were determined by titration on BHK cells. (**c**) Viral shedding was assessed via throat and cloacal swabs collected from infected ducks at 2, 3 and 4 dpi. Virus litres were determined by titration on BHK cells. (**d**) Detection of TMUV-specific antibodies in serum samples of inoculated ducks, contact ducks and non-infected ducks using ELISA. Seroconversion was defined by a PI value of ≥18.4%, indicating a positive antibody response to the virus.

To determine the infectivity of cluster 3 TMUV SD strain for ducks, both *in vitro* and *in vivo* experiments were conducted. The viral growth curves indicated that the TMUV SD replicates efficiently in DEF cells, with robust viral replication observed after infection at a MOI of 0.0001 (Fig. 1a) and a peak titre of 10^5.75^ TCID_50_ ml^−1^ at 36 hpi. *In vivo*, to assess the infection and transmission potential of TMUV SD among ducks, we measured the viral litres in blood, throat and cloacal swab samples from six inoculated ducks. The results revealed that nearly all inoculated ducks were viremic at 2 and 3 dpi (Fig. 1b). Viral shedding was detected in throat and cloacal swabs at 3 and 4 dpi, especially in throat swabs, with almost all infected ducks shedding virus through the throat (Fig. 1c). To directly see whether SD can be transmitted through direct contact, three naïve ducks were placed in the same cage 1 day after the initial ducks were inoculated. Antibody testing of the contact group showed that all three contact ducks were seropositive (Fig. 1d), indicating that the TMUV SD can indeed be transmitted through direct contact. In inoculated ducks, TMUV SD was detected in the heart, spleen, kidney, lung, duodenum, thymus gland and bursa of Fabricius of inoculated ducks at 3 dpi (Table 1), with the highest titre in the spleen around 10^6.11^ g^−1^. No virus was detected at 5 dpi.

**Table 1.** TMUV titres in organ/tissue samples of infected ducks tested on BHK cells.

Time points (dpi)	Virus titre (log_10_TCID_50_ g^−1^)*										
	Heart	Liver	Spleen	Lung	Kidney	Ovary	Duodenum	Pancreas	Thymus gland	Bursa of Fabricius	Brain
3	2.33/2.67/-	-/-/-	6.11±1.02	2.55±0.69	-/-/-	-/-/-	3.33±0.76	-/-/-	4.44±0.51	3.33/-/3.5	-/-/-
5	-/-/-	-/-/-	-/-/-	-/-/-	-/-/-	-/-/-	-/-/-	-/-/-	-/-/-	-/-/-	-/-/-

*Where the virus was detected in all three ducks sampled, the mean TCID_50_ ± the sd is shown; otherwise, individual titres are shown for each animal. –, No virus was detected.

### Cluster 3 Tembusu virus SD can infect chickens but cannot transmit directly between chickens

**Fig. 2.**
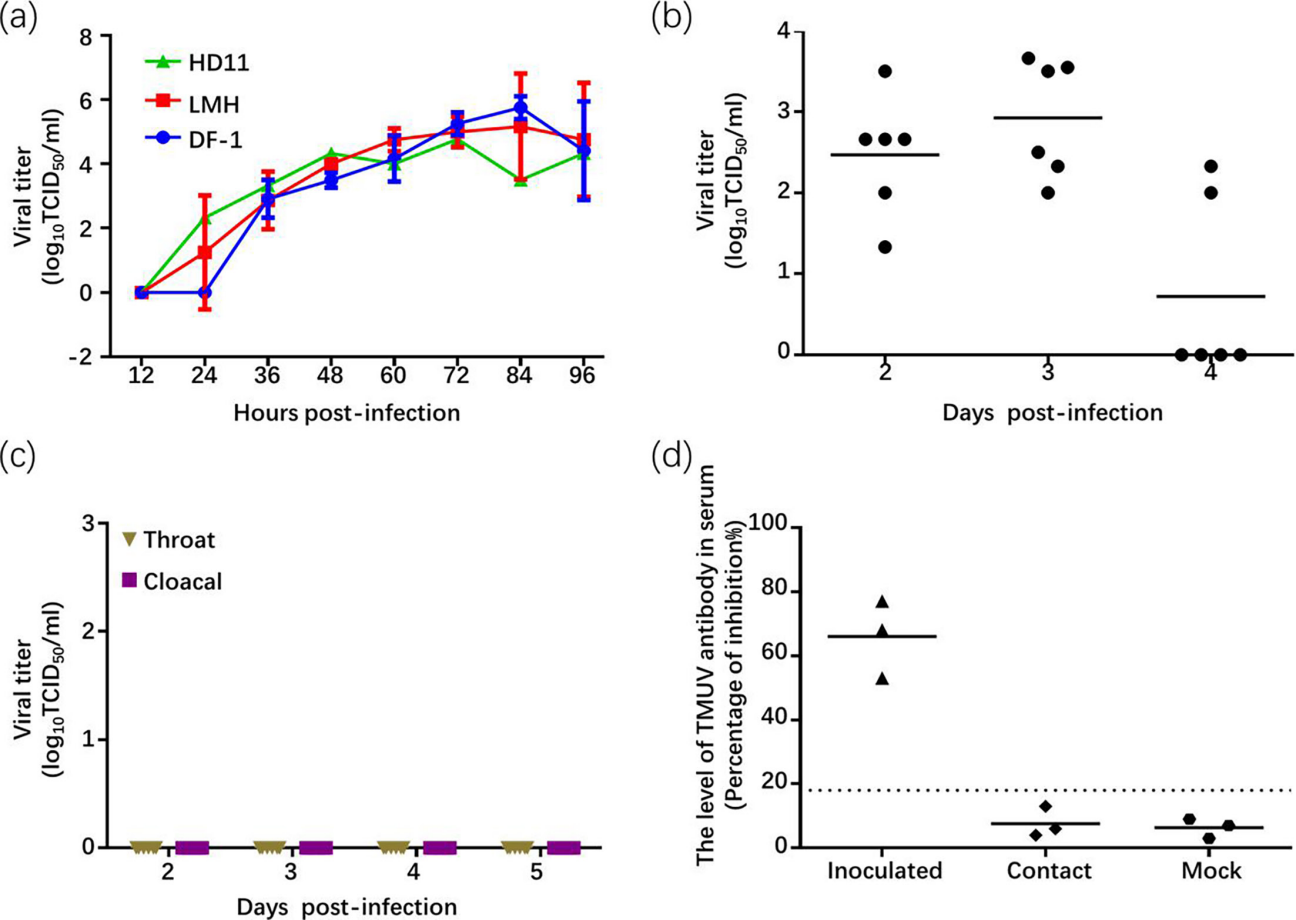
*In vitro* and *in vivo* results of cluster 3 TMUV infection in chickens. (**a**) Growth curves of TMUV SD on HD11, LMH and DF-1 chicken cell lines infected at a MOI of 0.0001. Supernatant samples were collected at designated time points and titrated on BHK cells to determine viral replication efficiency. (**b**) Viremia in infected chickens: Chickens were intramuscularly inoculated with 10^3.5^ TCID_50_ of the SD strain. The virus litres in blood collected at 2, 3 and 4 dpi were determined by titration on BHK cells. Results indicate viremia development at these time points. (**c**) Viral shedding detection: Virus litres were assessed in throat and cloacal swabs collected from infected chickens at 2, 3 and 4 dpi, and no virus was detected in all swab samples. (**d**) Detection of TMUV-specific antibodies in serum samples of inoculated chickens at 14 dpi, contact chickens and non-infected chickens using ELISA. Seroconversion was defined by a PI value of ≥18.4 %, indicating a positive antibody response to the virus.

To investigate whether TMUV SD can infect chickens, we conducted parallel experiments to those performed in ducks. The growth curve analysis demonstrated that TMUV SD replicates efficiently in three chicken cell lines: DF-1, LMH and HD-11, which represent fibroblasts, hepatocytes and immune cells, respectively, and reflect viral replication in different tissue-derived cells (Fig. 2a). To further investigate the replication and transmission potential of TMUV SD in chickens, we measured viral litres in blood, throat and cloacal swabs from six inoculated chickens. Viremia was detected in all chickens at 2 and 3 dpi (Fig. 2b), but no viral shedding was observed in the throat or cloacal swabs at 3 and 4 dpi (Fig. 2c). Anti-TMUV antibody testing in three chickens in the contact group, which were placed in the same cage 1 day after the initial chickens were inoculated, revealed that all three contact chickens remained negative (Fig. 2d). This indicates that cluster 3 TMUV does not transmit through direct contact among chickens. Virus replication in the inoculated chickens was observed in the liver, spleen, kidneys, duodenum and thymus gland at 3 dpi (Table 2), with the highest viral titre in the spleen. The virus was also isolated from the heart, ovary and lung in one or two of the three infected chickens.

**Table 2.** TMUV litres in organ/tissue samples of infected chickens tested on BHK cells

Time points (dpi)	Virus titre (log_10_TCID_50_/g)*										
	Heart	Liver	Spleen	Lung	Kidney	Ovary	Duodenum	Pancreas	Thymus gland	Bursa of Fabricius	Brain
3	-/-/2.5	2.83±1.06	3.5±1.08	1.33/-/1.5	2.33±0	-/-/3.5	1.77±0.31	-/-/-	2.83±0.23	-/-/-	-/-/-
5	-/-/-	-/-/-	-/-/-	-/-/-	-/-/-	-/-/-	-/-/-	-/-/-	-/-/-	-/-/-	-/-/-

*Where the virus was detected in all three ducks sampled, the mean TCID_50_ ± the sd is shown; otherwise, individual titres are shown for each animal. –, No virus was detected.

### Tembusu virus SD can infect *C. pipiens* mosquitoes

**Fig. 3.**
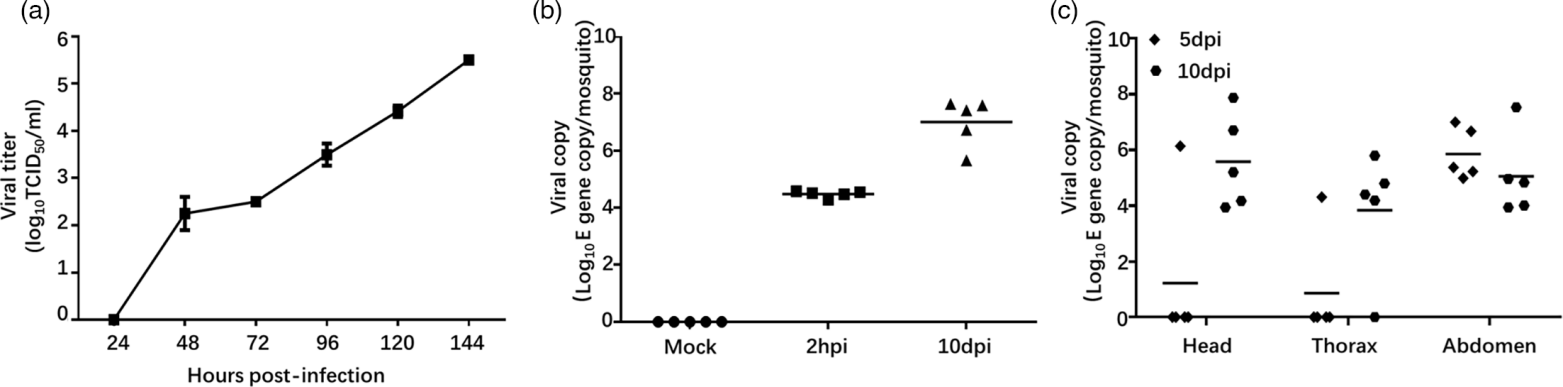
*In vitro* and *in vivo* infection of TMUV SD in *C. pipiens* mosquitoes. (**a**) Replication kinetics: growth curves of cluster 3 TMUV (SD strain) in C6/36 mosquito cells infected at an MOI of 0.0001. Supernatant samples were collected at various time points and titrated on BHK cells. (**b**) Viral load in mosquitoes: Chickens were intramuscularly inoculated with 10^3.5^TCID_50_ of the SD strain. The chickens were bitten by 4-day-old female *C. pipiens* mosquitoes at 3 dpi. Blood-engorged mosquitoes were selected and reared as the infected group. The viral E gene copy number in five mosquitoes was quantified by qPCR at 0 and 10 dpi. (**c**) Dissemination of TMUV SD in mosquito tissues: at 0 and 10 dpi, viral E gene copies were quantified in the head, thorax and abdomen of five infected mosquitoes using qPCR.

To assess whether mosquitoes can be infected by TMUV SD, we first established the virus growth curve in the mosquito cell line C6/36. The results indicate that the virus replicates efficiently in mosquito cells. To test whether *C. pipiens* mosquitoes can be infected with TMUV SD by biting infected chickens, we simulated natural mosquito biting conditions and allowed *C. pipiens* females to directly feed on chickens that had been infected with TMUV 3 days earlier and were expected to exhibit high viremia based on Fig. 2b. Subsequently, five mosquitoes were randomly sampled for viral load analysis, looking at both whole mosquitoes and in the main body parts: head, thorax and abdomen. qPCR was chosen for detecting viral replication and dissemination in mosquitoes, as their small size and the difficulty of thorough homogenization make nucleic acid quantification the most practical and sensitive approach. The analysis revealed that all five mosquitoes were positive for TMUV at 10 dpi, with an average of 1.01×10^7^ TMUV E gene copies per mosquito. Compared with around 10^4^ TMUV E gene copies after biting, the increased quantity of TMUV E gene copies indicates that the virus replicates in mosquitoes. No TMUV was detected in the control mosquitoes (mock-infected group) (Fig. 3b). At 10 dpi, the virus had disseminated from the gut to secondary tissues and was found in the head, thorax and abdomen, exhibiting variable viral loads (Fig. 3c).

**Fig. 4.**
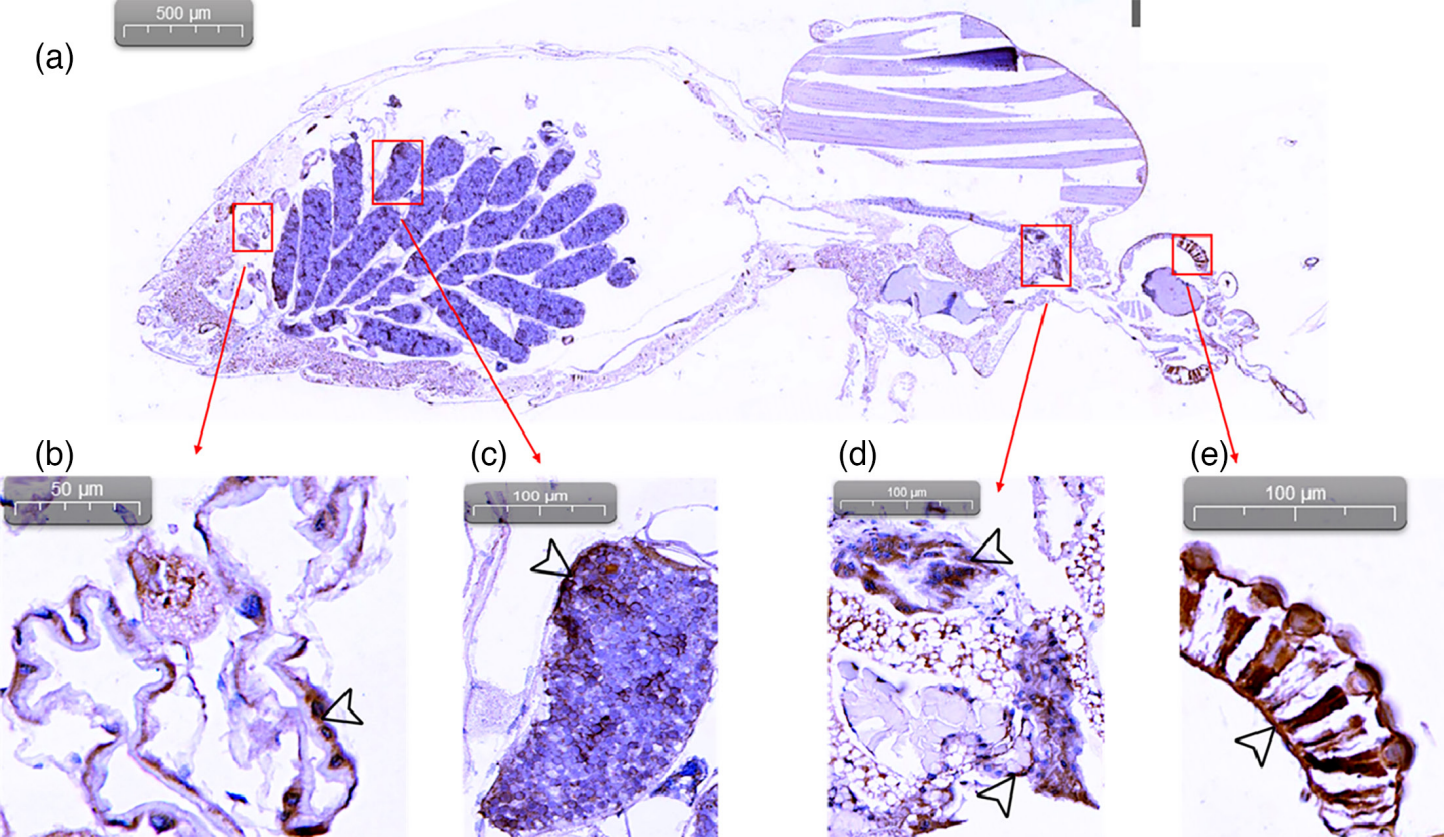
Immunohistochemical staining of TMUV SD in paraffin-embedded mosquitoes. (**a**) Overall view of an infected mosquito. (**b**) Infected midgut. (**c**) Infected ovary. (**d**) Infected salivary glands. (**e**) Infected ommatidia. TMUV SD antigen stains brownish black. The arrowhead points towards the positive region.

To detect the localization of TMUV antigen in infected mosquitoes, immunohistochemistry analysis was conducted on various tissues from infected mosquitoes (Fig. 4a). The tissues analysed included the midgut, salivary glands, ommatidia and ovaries, based on established protocols for West Nile Virus infection in mosquitoes [22]. Infected mosquito midgut (Fig. 4b), ovary (Fig. 4c), salivary glands (Fig. 4d) and ommatidia (Fig. 4e) exhibited a distinct brown stain, indicating the presence of TMUV antigen at 10 dpi. Notably, the salivary glands displayed pronounced brown staining, which strongly suggests that TMUV SD had successfully replicated and accumulated in these glands. This is a critical finding as it indicates the potential for *C. pipiens* mosquitoes to transmit TMUV to a new host through biting. The presence of TMUV antigen in the ommatidia and ovaries further suggests systemic infection within the mosquito, which could have implications for both vertical transmission and overall vector competence.

### TMUV SD needs mosquitoes to be transmitted among chickens

**Fig. 5.**
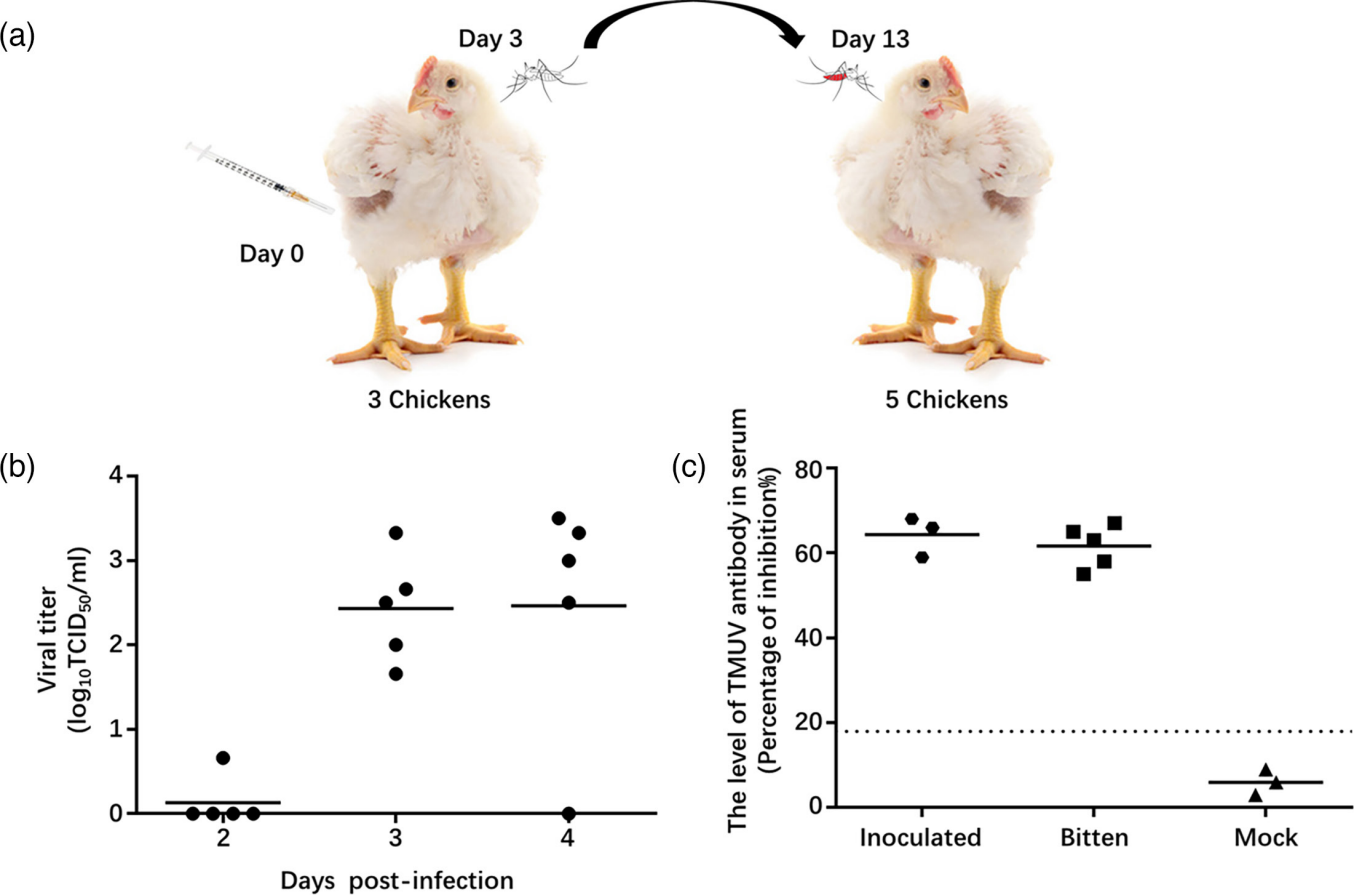
Transmission of TMUV SD in chickens via mosquito bites. (**a**) Schematic representation of the infection of chickens via mosquito bites. (**b**) The occurrence of viremia in chickens bitten by infected mosquitoes was detected by virus titration with the blood samples collected at 2, 3 and 4 days post-bites on BHK cells. (**c**) TMUV-specific antibody test: the TMUV-specific antibodies in the serum samples of three inoculated chickens, five bitten chickens, and three contact chickens were measured by ELISA after 2 weeks post-bites. A positive seroconversion is noted when the PI value exceeds 18.4%.

To investigate whether TMUV SD requires mosquitoes for transmission among chickens, we designed an experiment to simulate natural infection conditions (Fig. 5a). In this experiment, 50 mosquitoes were allowed to bite 3 chickens that had been intramuscularly inoculated with the SD virus for 3 days. After the bites, the mosquitoes were kept for 10 days, during which they were allowed to lay eggs for subsequent biting to be finished. After 10 days, these mosquitoes were used to bite five 1-month-old chickens. Three days after the bites, all five chickens developed viremia, confirming that the SD virus was successfully transmitted to the new chickens via the infected mosquitoes (Fig. 5b). Additionally, 2 weeks later, serological analysis showed that all five chickens had TMUV antibody-positive serum, indicating that the infection was successful and that an adaptive immune response was generated, compared to three other chickens that were not bitten by mosquitoes but were housed in the same cage (Fig. 5c). These findings demonstrate that mosquito vectors are necessary for TMUV transmission between chickens.

## Discussion

TMUV is a mosquito-borne flavivirus that has caused significant economic losses in the duck farming industry due to novel transmission routes, via airborne and direct contact mechanisms, in ducks [23]. Understanding the transmission pathways of TMUV in poultry is critical for developing targeted control strategies to mitigate its impact [16]. However, the transmission mechanisms of the newly isolated cluster 3 TMUV remain largely unexplored. This study provides both *in vitro* and *in vivo* evidence regarding the infection and transmission of cluster 3 TMUV in ducks and chickens.

Cluster 3 TMUV was initially isolated from mosquitoes, with the first poultry strain identified in ducks [12]. For years, there were few reports of cluster 3 infections in poultry until 2020, when outbreaks linked to a decline in egg production emerged across China, implicating the rise in cluster 3 strain circulation. Given that cluster 3 was first identified in ducks, it is important to first understand its pathogenicity in ducks and its transmission routes within this species. Our experiments demonstrate that the cluster 3 SD strain is capable of replicating in duck cells, and its replication is even more rapid than in chicken cells. Animal experiments also show that cluster 3 TMUV can infect various duck organs and, importantly, can be transmitted among ducks through direct contact. This transmission pattern mirrors that of the classic cluster 2 TMUV FX2010 strain, suggesting that cluster 3 could become widely prevalent in duck populations. The fact that the cluster 3 SD strain was not first isolated from ducks might be due to the widespread vaccination of ducks but not chickens. This raises concerns about the evolving complexity of TMUV outbreaks in China and poses new challenges for disease prevention and control.

Orthoflaviviruses, such as yellow fever, dengue and West Nile viruses, rely on mosquitoes for transmission, and controlling mosquito populations is an effective strategy for reducing the spread of these viruses. However, TMUV has evolved beyond this dependency, particularly in ducks, where it can spread rapidly regardless of the season [16]. Previous research indicated that the TMUV FX2010 strain can be efficiently transmitted among ducks without mosquitoes, whereas the oldest TMUV cluster 3 strain MM1775 isolated from mosquitoes in Malaysia in 1955 cannot transmit via direct contact among ducks [19]. Compared to FX2010, MM1775 cannot be isolated from the lungs of infected ducks, which explains why MM1775 cannot be transmitted through direct contact. However, cluster 3 TMUV can be isolated in low amounts from the infected chicken lungs but still fails to transmit directly in chickens. Yan *et al*. [12] reported that the cluster 3 TMUV SD strain cannot transmit directly in chickens but can infect via intranasal routes, indicating that this virus can be transmitted through the respiratory tract. Our research shows that even though cluster 3 TMUV is present in low amounts in the lungs, it still cannot shed the virus through the respiratory tract, and this appears to be the main reason why it cannot transmit through direct contact in chickens.

TMUV was first identified in *C. tritaeniorhynchus* mosquitoes in 1955 [1] and later in *Culex gelidus* and *Culex vishnui* in Southeast Asia [24,25]. In China, TMUV has been detected in *C. tritaeniorhynchus* mosquitoes in Yunnan Province and Chongming Island, with both strains belonging to cluster 3 [26]. However, direct experimental evidence shows that cluster 3 TMUV can be transmitted by *Culex* spp. mosquitoes to chickens has been lacking. Our study is the first to demonstrate that cluster 3 TMUV can indeed be transmitted from chicken to chicken by *Culex* mosquitoes (*C. pipiens* was tested) while taking a blood meal. These mosquito transmission data provide critical new insights into cluster 3 TUMV transmission dynamics.

## Conclusion

**Fig. 6.**
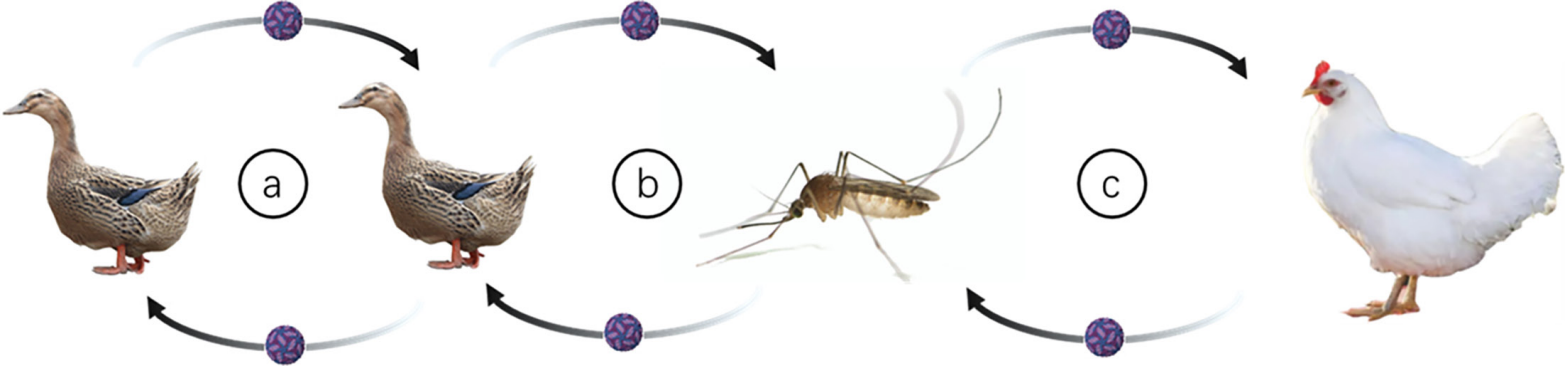
Transmission dynamics of cluster 3 TMUV. (**a**) Cluster 3 TMUV SD can be transmitted among ducks through direct contact. (**b**) Cluster 3 TMUV SD can be transmitted among ducks by mosquito vectors. (**c**) Mosquito vectors are essential for the transmission of cluster 3 SD TMUV.

This study provides valuable insights into the transmission dynamics of cluster 3 TMUV in both ducks and chickens (Fig. 6). We demonstrated that cluster 3 TMUV SD can be efficiently transmitted among ducks through direct contact, bypassing the need for mosquito vectors, which mirrors the transmission patterns of earlier cluster 2 TMUV strains like FX2010. However, in chickens, mosquito vectors remain essential for the transmission of cluster 3 TMUV. Our findings also highlight the potential role of the respiratory tract and digestive tract excretion in the spread of TMUV.

## Abbreviations

AMV: Avian Myeloblastosis Virus
BHK: baby hamster Syrian kidney
DEF: duck embryo fibroblast
dpi: days post-inoculation
hpi: hours post-infection
MOI: multiplicity of infection
PI: percent inhibition
qPCR: quantitative real-time PCR
SD: Shandong, 2021
TMUV: Tembusu virus

## References

[1] Platt GS, Way HJ, Bowen ETW, Simpson DIH, Hill MN (1975). Arbovirus infections in Sarawak, october 1968--february 1970 *Tembusu* and *Sindbis* virus isolations from mosquitoes. Ann Trop Med Parasitol.

[2] Kono Y, Tsukamoto K, Abd Hamid M, Darus A, Lian TC (2000). Encephalitis and retarded growth of chicks caused by *Sitiawan* virus, a new isolate belonging to the genus *Flavivirus*. Am J Trop Med Hyg.

[3] O’Guinn ML, Turell MJ, Kengluecha A, Jaichapor B, Kankaew P (2013). Field detection of *Tembusu* virus in western Thailand by rt-PCR and vector competence determination of select culex mosquitoes for transmission of the virus. Am J Trop Med Hyg.

[4] Yan P, Zhao Y, Zhang X, Xu D, Dai X (2011). An infectious disease of ducks caused by a newly emerged *Tembusu* virus strain in mainland China. Virology.

[5] Homonnay ZG, Kovács EW, Bányai K, Albert M, Fehér E (2014). *Tembusu*-like flavivirus (*Perak virus*) as the cause of neurological disease outbreaks in young pekin ducks. Avian Pathol.

[6] Thontiravong A, Ninvilai P, Tunterak W, Nonthabenjawan N, Chaiyavong S (2015). *Tembusu*-related *Flavivirus* in ducks, Thailand. Emerg Infect Dis.

[7] Su J, Li S, Hu X, Yu X, Wang Y (2011). Duck egg-drop syndrome caused by BYD virus, a new *Tembusu*-related *Flavivirus*. PLoS One.

[8] Hamel R, Phanitchat T, Wichit S, Morales Vargas RE, Jaroenpool J (2021). New insights into the biology of the emerging *Tembusu* Virus. *Pathogens*.

[9] Wang X, Chen X, Ding Y, Xu P, Wang C (2025). Isolation, identification, and pathogenicity evaluation of a novel cluster 3 *Tembusu virus* isolated from geese in China. Poult Sci.

[10] Cui Y, Pan Y, Guo J, Wang D, Tong X (2022). The evolution, genomic epidemiology, and transmission dynamics of *Tembusu virus*. Viruses.

[11] Lei W, Guo X, Fu S, Feng Y, Tao X (2017). The genetic characteristics and evolution of *Tembusu virus*. Vet Microbiol.

[12] Yan D, Li X, Wang Z, Liu X, Dong X (2022). The emergence of a disease caused by a mosquito origin cluster 3.2 *Tembusu virus* in chickens in China. Vet Microbiol.

[13] Liao J-Y, Tang H, Xiong W-J, Liu T-N, Li J-Y (2023). Isolation and characterization of a novel goose ovaritis-associated cluster 3 *Tembusu virus*. Poult Sci.

[14] Yu Z, Ren H, Sun M, Xie W, Sun S (2022). *Tembusu virus* infection in laying chickens: evidence for a distinct genetic cluster with significant antigenic variation. Transbound Emerg Dis.

[15] Fang Y, Hang T, Yang L-M, Xue J-B, Fujita R (2023). Long-distance spread of *Tembusu virus*, and its dispersal in local mosquitoes and domestic poultry in Chongming Island, China. Infect Dis Poverty.

[16] Li X, Shi Y, Liu Q, Wang Y, Li G (2015). Airborne transmission of a novel *Tembusu virus* in ducks. J Clin Microbiol.

[17] Liu X, Yan D, Peng S, Zhang Y, Xu B (2023). 326K at E protein is critical for mammalian adaption of TMUV. Viruses.

[18] Habjan M, Penski N, Spiegel M, Weber F (2008). T7 RNA polymerase-dependent and -independent systems for cDNA-based rescue of rift valley fever virus. J Gen Virol.

[19] Yan D, Shi Y, Wang H, Li G, Li X (2018). A single mutation at position 156 in the envelope protein of *Tembusu virus* is responsible for virus tissue tropism and transmissibility in ducks. J Virol.

[20] Yan L, Yan P, Zhou J, Teng Q, Li Z (2011). Establishing a TaqMan-based real-time PCR assay for the rapid detection and quantification of the newly emerged duck *Tembusu virus*. Virol J.

[21] Li X, Li G, Teng Q, Yu L, Wu X (2012). Development of a blocking ELISA for detection of serum neutralizing antibodies against newly emerged duck *Tembusu Virus*. PLoS One.

[22] Girard YA, Klingler KA, Higgs S (2004). West Nile virus dissemination and tissue tropisms in orally infected *Culex* pipiens quinquefasciatus. Vector Borne Zoonotic Dis.

[23] Tunterak W, Ninvilai P, Tuanudom R, Prakairungnamthip D, Oraveerakul K (2021). Evaluation of host systems for efficient isolation and propagation of duck *Tembusu virus*. Avian Pathol.

[24] Pandey BD, Karabatsos N, Cropp B, Tagaki M, Tsuda Y (1999). Identification of a *Flavivirus* isolated from mosquitos in Chiang Mai Thailand. Southeast Asian J Trop Med Public Health.

[25] Leake CJ, Ussery MA, Nisalak A, Hoke CH, Andre RG (1986). Virus isolations from mosquitoes collected during the 1982 Japanese encephalitis epidemic in northern Thailand. Trans R Soc Trop Med Hyg.

[26] Hu D, Wu C, Wang R, Yao X, Nie K (2023). Persistence of Tembusu Virus in *Culex tritaeniorhynchus* in Yunnan Province, China. Pathogens.

